# Detection of Coxsackievirus A24v during an acute haemorrhagic conjunctivitis outbreak in Dar es Salaam, Tanzania, January-February 2024

**DOI:** 10.1371/journal.pone.0352698

**Published:** 2026-06-25

**Authors:** Lawrence A. Mapunda, Peter van Heusden, Robert Baluhya, Olympia G. Machange, Ambele Mwafulango, Monica Fredrick Francis, Aziz Ituka, Omega Machange, Adela Kisanga, Edna E. Mgimba, Hamza H. Matimba, Dennis M. Kado, Elson E. Kimaro, Ramadhani A. Libenanga, Jackson Peter Mushumbusi, Hamisi M. Swalehe, Ibrahim Mauki, James Hellar, Shimba Henerico, Maria E. Kelly, Nyambura Moremi

**Affiliations:** 1 Tanzania National Public Health Laboratory (NPHL), Dar es Salaam, Tanzania; 2 South African Medical Research Council Bioinformatics Unit, South African National Bioinformatics Institute, University of the Western Cape, Bellville, South Africa; 3 Tanzania Ministry of Health, Dodoma, Tanzania; 4 Bugando Medical Centre, Mwanza, Tanzania; 5 World Health Organization, Tanzania Country Office, Dar es Salaam, Tanzania; Institut Pasteur, FRANCE

## Abstract

**Background:**

In January 2024, an outbreak of acute viral conjunctivitis was declared in Dar es Salaam, Tanzania, following initial case clusters reported in December 2023 and continuing through February. Patients presented with red, itchy, and burning eyes, eyelid swelling, photophobia, and eye discharges. As the responsible pathogen had not been laboratory-identified, this study aimed to detect and characterize potential viral agents of the outbreak through an urgent outbreak response investigation.

**Methods:**

Conjunctival swabs were collected from 25 suspected cases using a convenience sampling approach. Bacterial culture was performed on MacConkey and blood agar. Viral detection used a multiplex real-time RT-PCR targeting adenovirus, metapneumovirus, enterovirus (EV), and parainfluenza virus. All enterovirus-positive samples with a Ct value <30 (four samples) were selected for genomic sequencing on an Illumina MiSeq platform. Maximum-likelihood phylogenetic analysis was conducted with IQ-TREE.

**Results:**

Nine of 25 samples (36%) were positive for enterovirus (EV) by real-time reverse transcription PCR. Genomic sequencing of the four eligible samples (Ct < 30) detected Coxsackievirus A24 variant (CVA24v) in two of four sequenced samples. Phylogenetic analysis showed that the Tanzanian outbreak sequences formed a clade with CVA24v strains from a concurrent 2024 outbreak in France/Mayotte and were closely related to recent sequences from East Africa and South America. Bacterial culture yielded no significant pathogens.

**Conclusion:**

We detected enterovirus (EV) during an outbreak of acute hemorrhagic conjunctivitis in Tanzania. Of nine EV-positive samples, CVA24v genomes were reconstructed from genomic sequencing in two samples, with phylogenetic analysis placing Tanzania sequencences within a clade with sequences from France (Mayotte), Mexico, Brazil, and Uganda strains consistent with regional circulation. Given that sequencing was performed on only two samples, a causal role for CVA24v in this outbreak cannot be definitively established; however, these findings provide the first genomic evidence of CVA24v circulation in Tanzania and demonstrate the value of rapid genomic epidemiology for outbreak pathogen detection in resource-limited settings.

## Introduction

Acute hemorrhagic conjunctivitis (AHC) is a highly contagious viral conjunctivitis caused primarily by human enteroviruses, Coxsackievirus A24v variant and Enterovirus 70, both single-stranded, non-enveloped RNA viruses of the Picornaviridae family [1]. Other human enteroviruses cause a wide range of illnesses, from mild respiratory and gastrointestinal syndromes to severe diseases such as poliomyelitis (Enterovirus C) [2,3], myocarditis (Coxsackievirus B) [4], and aseptic meningitis (echovirus) [5].

AHC occurs in explosive epidemics, particularly in tropical and subtropical regions with high humidity and dense populations [6]. Although infections are self-limiting, they often disrupt schools, workplaces, and community productivity. In healthcare facilities, sudden surges in AHC cases strain clinical and laboratory capacity, temporarily diverting resources from essential health services [6].

Typical symptoms include sudden onset of ocular pain, foreign-body sensation, eyelid oedema, subconjunctival hemorrhage, tearing, and photophobia, which resolve within one to two weeks [7]. Fluids from an infected person’s eyes are highly contagious and spread from person to person through hand-to-eye-to-hand contact and contaminated fomites, making outbreaks difficult to contain once community transmission begins.

AHC was first identified in Ghana in 1969 [8] and subsequently spread across West Africa, Southeast Asia, and the world. The first large epidemic in the Western Hemisphere occurred in 1981 [7]. Since then, outbreaks have been reported in 15 of the 21 Global Burden of Disease regions. In East Africa, recurrent epidemics have been documented in Kenya, Uganda, and Tanzania, typically during humid seasons, though laboratory confirmation of etiologic agents has often been limited by diagnostic capacity [9]. In Kenya, Enterovirus 70 was first isolated in 1971 and during a second outbreak in 1974. Coxsackievirus A24v etiology was confirmed in Uganda and South Sudan during a conjunctivitis outbreak in 2010. Likewise, Dar es Salaam has experienced recurrent AHC outbreaks since the early 1980s, including one in 2010 [10]. However, due to limited diagnostic capacity, responsible pathogens were not conclusively identified and confirmed [10].

In December 2023, clinicians in Dar es Salaam, Tanzania, reported clusters of patients presenting with acute red eye, pain, tearing, and subconjunctival hemorrhage [11]. On January 15, 2024, the Ministry of Health investigation reported an outbreak of acute viral conjunctivitis, locally known as “red-eye disease.” By January 11, 2024, reported cases in Dar es Salaam had increased from 17 to 869, indicating rapid spread [11]. The outbreak then expanded swiftly, and by January 26, cases had been reported in 17 of Tanzania’s 30 regions—Singida, Katavi, Kilimanjaro, Mara, Iringa, Njombe, Ruvuma, Simiyu, Mtwara, Lindi, Songwe, Rukwa, Mwanza, Dar es Salaam, Morogoro, Dodoma, and Pwani—with cumulative cases rising from 1,109–5,359 within one week [12]. Because data included only symptomatic individuals seeking care, the true burden in the community was stated by the Ministry of Health to be likely higher.

At the onset of this investigation, the etiologic agent was unknown. Public health leaders required urgent laboratory investigation to guide response measures. To our knowledge, no previous genomic characterization of the pathogen responsible for AHC outbreaks in Tanzania has been reported, despite viral conjunctivitis outbreaks being documented in Dar es Salaam since the early 1980s [10]. This study therefore aimed to rapidly detect and characterize potential viral agents of the acute conjunctivitis outbreak in Dar es Salaam between January and February 2024.

## Methods

### Study design and sampling strategy

This investigation was conducted as an urgent outbreak response following the Ministry of Health’s declaration of a suspected viral conjunctivitis outbreak on January 15, 2024. The primary objective was to rapidly identify and characterize the etiologic agent to inform public health interventions, not to conduct a population-based epidemiological study. Consequently, a convenience sampling approach was employed.

A total of 25 conjunctival swab pairs were collected between February 5 and 8, 2024, from patients presenting with acute conjunctivitis at several clinics in Dar es Salaam. Patients presenting with clinical signs consistent with acute viral conjunctivitis (redness, itching, burning, eyelid swelling, photophobia, or eye discharge) during the outbreak period were investigated.

No sample size calculation was performed, as the goal was rapid etiologic confirmation rather than estimation of population-level prevalence or generalizability. Sampling was limited by logistical constraints, including: (i) the acute nature of the outbreak requiring immediate laboratory action, and (ii) the need to prioritize speed over comprehensive geographic representation. All samples were collected from Dar es Salaam, where the outbreak was first reported and most intensely active at the time of investigation.

Ethics statement: This study complies with the principles stated in the Declaration of Helsinki (Ninth Revision, October 2013) and relevant national and regulatory guidelines. The study protocol was submitted to the National Health Research Ethics Committee (NatHREC) for review and approval and was granted certificate NIMR/HQ/R.8a/Vol.IX/4916. Authorization to conduct the study was obtained from the National Public Health Laboratory management. Patient identifying information has been anonymized to preserve privacy; only necessary information was collected during sample collection. The samples analyzed in this investigation were collected as part of Ministry of Health outbreak response activities to the acute viral conjunctivitis outbreak. In Tanzania, as stated in the Public Health Act, 2009 (Section 45), informed consent is not required for specimens collected during declared public health emergencies for the purpose of diagnostic investigation and outbreak response [13].

In Tanzania, as stated in the Public Health Act, 2009 (Section 11 and section 45), informed consent is not required for specimens collected during declared public health emergencies for the purpose of diagnostic investigation and outbreak response. The authors had access to information that could identify individual participants during data collection. For research purposes medical records were accessed between 11/04/2025 and 18/04/2025. Following data extraction, the data set was anonymized, and all personal identifiers were removed to ensure participant confidentiality. All the analyses were performed on de-identified datasets.

### Sample collection

Paired conjunctival swab samples were collected from 25 patients who presented with acute conjunctivitis at several clinics in Dar es Salaam. One swab, placed in viral transport medium (VTM), was used for real-time RT-PCR and metagenomic sequencing, and the other, in Stuart transport medium, for bacterial culture. Samples were collected between February 5 and 8, 2024, and immediately transported to the National Public Health Laboratory (NPHL) for analysis. Samples in Stuart transport medium were transported at ambient temperature, whereas those in VTM were maintained under cold-chain conditions (2–8°C).

### Laboratory characterization

#### Bacterial culture.

For bacteriological analysis, conjunctival swabs from each of the 25 cases were inoculated onto MacConkey and blood agar, incubated at 37°C in 5% CO₂ for 18 hours, and examined for growth. Bacterial identification was performed using matrix-assisted laser desorption ionization time-of-flight (MALDI-TOF) mass spectrometry on the Vitek MS system.

#### RNA extraction.

Samples for real-time RT-PCR molecular detection underwent RNA extraction using the QIAamp Mini RNA Kit (Qiagen, Germany) according to the manufacturer’s instructions.

#### Real-time RT-PCR.

We performed quantitative reverse transcriptase polymerase chain reaction (real-time RT-PCR) using Seegene’s Allplex respiratory panel 2 (Seegene, South Korea), which targets adenovirus, metapneumovirus, (EV), and parainfluenza virus, according to the manufacturer’s instructions, on the CFX96 Biorad and the results were interpreted using the Seegene software.

Extracted RNA from four EV-positive samples with cycle threshold below 30 were converted to cDNA using the Illumina’s cDNA synthesis kit and then subjected to genomic sequencing using the Illumina MiSeq platform (Illumina). The sequencing library was prepared using Illumina’s Respiratory Pathogen ID/AMR enrichment panel kit (RPIP kit) according to the manufacturer’s instructions.

FASTQ reads were uploaded to the usegalaxy.eu Galaxy server [14] and quality-trimmed using Fastp [15]. Host reads were removed by mapping to the Hg38 Homo sapiens genome assembly as previously described [16]. Analysis with Kraken2 [17] with the Standard-Full database (dated 2022-06-07) confirmed the presence of reads matching Enterovirus C and D in three samples (28A0, 26HB, and 25AM) out of four sequenced samples. Enterovirus D reads detected in sample 25AM were further examined; BLAST analysis of the assembled contigs from this sample against the NCBI Virus database did not yield a high-confidence match to a complete Enterovirus D genome, and the detection is therefore considered a low-level, possibly artefactual finding that does not alter the primary conclusions of this study. One sample (27YS) showed the presence of *Streptococcus pneumoniae*, a known cause of bacterial conjunctivitis [18].

Samples 28A0, 26HB, and 25AM were assembled with rnaviralSPAdes [19]. Full-length genomes of Enterovirus C and D were downloaded from NCBI Virus (National Library of Medicine (US), National Center for Biotechnology Information, 2024) (1,982 genomes) and searched using BLAST [20,21] with the assembled contigs from the three viral samples as queries. Two of the three samples (28A0 and 26HB) yielded high-quality hits against a Coxsackievirus A24 genome, PP548240, a genome sampled in the French overseas Department of Mayotte and assembled from Oxford Nanopore sequencing.

Quality-trimmed reads from samples 28A0 and 26HB were aligned against MG880745.2 (a genome sampled in Mexico and assembled using Sanger sequencing), and variants were called using snippy [22]. Consensus genomes were constructed by inserting the variants called by snippy into the MG880745.2 reference genome and masking regions with low coverage with Ns using mosdepth and bcftools [23,24]. This remapping and variant-calling step was performed to generate high-quality consensus sequences from the assembled contigs, enabling accurate SNP calling relative to a well-characterized reference genome and ensuring reliable downstream phylogenetic placement.

#### Viral typing and phylogenetic analysis.

Typing with the RIVM Enterovirus typing tool [25] identified both consensus sequences as belonging to the A24v variant of Coxsackievirus A24 with the VP1 region assigned to genotype IV. Based on this classification, 69 full-length genome sequences of Coxsackievirus A24 of which both country and date of isolation were available were downloaded from NCBI Virus and classified using the RIVM Enterovirus typing tool. Viruses not classified as A24v (EF026081 and KU183495) were excluded from analysis, yielding a set of 67 full-length genome sequences. This set of genome sequences was combined with the two consensus genomes, and a multiple sequence alignment was computed using MAFFT [26]. D90457.1, the prototype strain of A24v (24/70) collected in Singapore in 1970, was selected as an outgroup and a maximum likelihood tree was constructed using IQ-TREE [27] with the GTR + F + I + G4 model selected by the Model Finder according to the Bayesian Information Criteria and 1000 bootstrap replicates. Tips and ancestral nodes were dated using the LSD2 method [28] built into IQ-TREE2 and collected date information for all sequences in the analysis.

As many more partial Coxsackievirus A24 genome sequences are available than full-length genome sequences, 1,967 partial genome sequences were downloaded from NCBI Virus. The EF026081 full-length sequence of the 1952 Coxsackievirus A24 Joseph strain was used to build a model for VADR [29] and this was used to annotate all full-length and partial Coxsackievirus A24 sequences. The VP1 sequence was extracted from both full-length and partial sequences based on VADR annotation. Partial genome sequences were selected for inclusion based on availability of date and country metadata, date greater than 1988 (for partial genome sequences), VP1 length greater than 900 base pairs and assignment to the A24v strain by the RIVM Enterovirus typing tool. This yielded a collection of 309 sequences (including the two newly generated consensus sequences). A multiple sequence alignment for all the VP1 sequences was computed using MAFFT, and a maximum likelihood tree was constructed using IQ-TREE.

A comparison between the tree generated from the full genome sequences and that generated from VP1 regions showed that the full genome tree was a subset of the VP1 tree and did not yield different phylogenetic results. Single nucleotide polymorphisms (SNPs) were extracted from the sequences using SNP-sites version 2.5.1, then the SNP distances were calculated using SNP-Dists version 1.2.0.

## Results

### Demographic and sampling characteristics

A total of 25 patients with acute conjunctivitis were enrolled from four districts of Dar es Salaam: Temeke (n = 9, 36%), Ubungo (n = 7, 28%), Ilala (n = 5, 20%), and Kigamboni (n = 4, 16%). The mean age of patients was 30 years, and 20/25 (80%) were male. No further demographic information about the patients was provided.

### Bacterial culture results

Of 25 samples cultured, 17 (68%) had no growth, while 7 (28%) samples had *Staphylococcus epidermidis* which is a commensal of the normal conjunctival flora, and 1 sample (4%) yielded *Bacillus cereus*. Bacillus cereus is a ubiquitous environmental organism that is also commonly found in the normal conjunctival microbiota; its isolation from a single sample in this study is therefore not considered clinically significant and does not indicate bacterial conjunctivitis as a contributing cause of this outbreak.

### Real-time RT-PCR results

Nine of 25 samples (36%) were positive for EV by real-time RT-PCR. Four samples with Ct values <30 were selected for genomic sequencing (Table 1 and Table 2).

**Table 1:** Enterovirus-positive clinical samples: cycle threshold (Ct) values by detection channel and internal control.

Well	Sample ID	Sequencing ID	EV Ctᵃ (Cal Red 610)	IC Ctᵇ (Quasar 670)
A01	S01	28A0	23.28	25.19
A03	S017	26HB	23.61	25.72
G01	S007	25YS	24.85	25.43
H03	S024	25AM	28.55	25.41
G02	S015	NA	32.98	25.64
C01	S003	NA	33.04	24.75
H02	S016	NA	33.24	25.57
C02	S011	NA	35.76	24.94
E01	S005	NA	36.17	24.78

ᵃ EV: Enterovirus. Samples are sorted in ascending order of EV Ct value (i.e., descending viral load). Nine of 24 clinical samples (37.5%) were EV-positive; Ct values ranged from 23.28 to 36.17, and no other target was amplified in clinical samples. Samples with Ct ≤ 30 (Sample 01, S017, S007, S024) selected for genomic sequencing. Sixteen of 24 clinical samples (62.5%) showed no amplification in any target channel. Internal controls were valid in all wells, ruling out extraction failure.

ᵇ IC: Internal control. IC Ct values were consistent across all positive wells (range 24.75–25.72), confirming adequate nucleic acid extraction and absence of PCR inhibition.

Abbreviations: Ct, cycle threshold; EV, Enterovirus; IC, internal control; PCR, polymerase chain reaction.

**Table 2:** Run control performance for the Allplex™ Respiratory Panel 2 assay.

Well	Name	Control type	Ct values	Outcome
F12	NTC	No-template control	All targets: undetermined; IC Ct: 36.38	Expected†
G12	NEG	Negative control	All targets: undetermined; IC Ct: 25.61	Expected†
H12	POS	Positive control	MPV: 27.34; PIV2: 26.83; PIV1: 33.30; AdV: 23.74; EV: 24.86; PIV3: 22.98; IC: 30.03	Expected‡

† Negative controls (NTC and NEG) correctly showed no amplification across all target channels. The “Invalid” software flag is applied by default to negative control wells by the Allplex™ system and does not indicate a failed run.

‡ The positive control (POS) amplified all expected targets. The “Invalid” flag is expected for positive control wells containing multiple targets, as the instrument’s interpretation algorithm is optimised for single-pathogen clinical specimens.

Abbreviations: AdV, Adenovirus; Ct, cycle threshold; EV, Enterovirus; IC, internal control; MPV, Metapneumovirus; NTC, no-template control; PIV, Parainfluenza virus.

All results were interpreted per the Seegene Allplex™ Respiratory Panel 2 Instructions for Use.

### Sequencing results

Two consensus sequences were generated (28AO and 26HB) with 84% and 64% of the 7,436 base pair reference genome (MG880745.2) covered at depth of 10 or more bases (bases with less than 10 × coverage were marked as N). Each sample differed from the reference genome by 252 and 242 SNPs, respectively. Comparison between the two Tanzanian sequences (28AO and 26HB) showed that they differed from each other by 22 SNPs, with no insertions or deletions detected relative to each other (**Table 3**).

**Table 3:** SNP difference between samples 26HB and 28AO and the reference MG88075.

	26HB	28AO	MG88075.2
**26HB**	0	22	242
**28AO**	22	0	252
**MG88075.2**	242	252	0

### Phylogenetic analysis results

**Fig 1:**
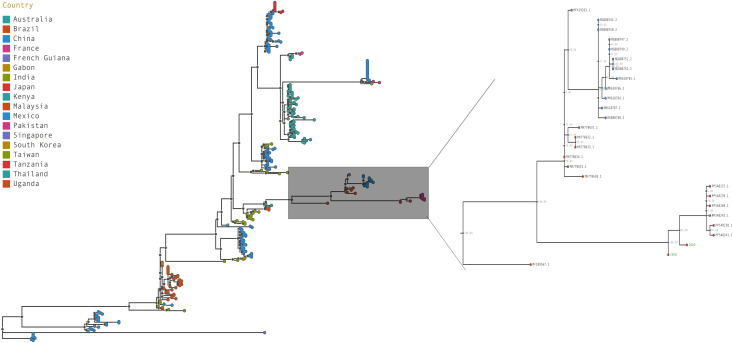
Phylogenetic tree built from VP1 sequences of Coxsackievirus A24v. The tree was generated using the maximum likelihood method with 1,000 bootstrap replicates. Tanzanian outbreak sequences (28AO and 26HB) are highlighted in bold and belong to Clade A, which includes sequences from East Africa and South America. Clade B comprises sequences predominantly from Asia. The prototype strain D90457.1 (Singapore, 1970) was used as an outgroup.

The phylogenetic tree built from both whole genome sequence and the VP1 sequences showed that the samples collected in the outbreak (28AO and 26HB) belong to a clade including sequences from several countries (Uganda, Mexico, Brazil, the French overseas Department of Mayotte and Tanzania) as well as South American countries (Brazil, Mexico and French Guiana) (**Fig 1 and** **S1 Fig**). This clade is distinct from another clade of virus circulating in several Asian countries (India, China, Thailand and others).

## Discussion

### Key findings

This investigation, conducted as an urgent outbreak response, provides the first genomic evidence of CVA24v detection during an acute hemorrhagic conjunctivitis outbreak in Tanzania. CVA24v was detected by genomic sequencing in two of four sequenced EV-positive samples, with phylogenetic analysis placing both sequences within a clade of contemporaneous CVA24v strains from East Africa and South America. While these findings are consistent with CVA24v as a likely contributor to this outbreak, a definitive causal attribution cannot be established on the basis of two genome sequences from a convenience sample of 25 cases. The high proportion of EV-negative samples (64%) and the absence of sequencing data for the remaining EV-positive samples further limit the conclusions that can be drawn. Prior AHC outbreaks in Dar es Salaam dating back to the 1980s were suspected to be viral in etiology but were never confirmed through genomic characterisation due to limited diagnostic capacity [10]; the current findings do not allow retroactive conclusions about the etiology of those earlier events.

Of the 25 convenience-sampled cases, 9 (36%) tested positive for EV. These findings are consistent with a 2010 study by the Centers for Disease Control and Prevention (CDC), which investigated a red eye syndrome outbreak in Uganda and South Sudan. In Uganda, 14 of 29 samples (48%) tested positive for enterovirus, while in South Sudan, 3 of 6 samples (50%) were positive. The same study indicated that 52% of Ugandan samples were negative for enterovirus [30], similar to the 64% negative rate observed in our investigation. In Madagascar, 55 specimens collected between January 10 and February 22, 2024, were analyzed at the Virology Unit of the Institut Pasteur, of which 18 (32.7%) tested positive for enterovirus [31]. It is worth noting that laboratory-negative results are common in viral conjunctivitis outbreaks and may be attributable to factors such as the timing of specimen collection [30,32].

### Phylogenetic and epidemiologic context

Samples collected in this outbreak belong to a clade that includes samples from South America (Brazil, Mexico, and French Guiana), as well as East Africa and its surrounding islands (Kenya in 2010, Uganda in 2017, and Mayotte in 2024). While the overall phylogeny constructed from recent Coxsackievirus A24v VP1 sequences displays a ladder-like evolutionary pattern, this clade is distinct from another clade comprising samples collected over the past decade in China, the Philippines, Thailand, and Australia Viruses closely related to two isolates of this 2024 Dar es Salaam outbreak have been circulating in the East African region and associated oceanic territories in recent years, though the limited number of genomes recovered precludes detailed inference of transmission dynamics.

Red eye conjunctivitis outbreaks have been associated with water, sanitation, and hygiene (WASH) practices [6]. Conjunctivitis outbreaks usually occur under favorable conditions such as hot, humid weather and crowded areas, facilitated by contaminated materials and water [33]. Significant flooding occurred in several Tanzanian regions between November and December 2023, including Arusha, Kigoma, Kagera, the Coast Region, Zanzibar, and most notably, Dar es Salaam. Dar es Salaam, a major commercial center in Eastern and Central Africa, faces challenges of overcrowding and inadequate sanitation facilities, which are overwhelmed during rainy seasons. Inhabitants frequently encounter open sewage systems, leading to a polluted environment and contaminated runoff water [34]. These factors increase the risk of diseases transmitted through fecal contamination of hands, fomites, and water [33]. Therefore, although heavy flooding occurred in late 2023 in many areas in the east African region, potentially contributing to transmission conditions [6,33], causal inference cannot be established.

### Limitations

We acknowledge important limitations regarding sample representativeness. Of the 25 samples, 80% were from males, and all were collected exclusively from Dar es Salaam despite the outbreak rapidly involving 17 regions by late January 2024. This reflects the convenience-based, urgency-driven nature of outbreak response sampling. The primary aim was not to achieve a population-representative sample but to obtain laboratory confirmation of the causative agent to guide public health action. At the time of sample collection (February 5–8, 2024), the etiology of the outbreak was unknown, and public health leaders required rapid answers.

The overrepresentation of males in our sample does not necessarily reflect the true sex distribution of cases in the community. It may instead reflect health-seeking behavior, occupational exposure, or sampling biases at the selected clinics. Similarly, while the outbreak spread to 17 regions, our sampling was necessarily concentrated in Dar es Salaam—the epicenter and site of the initial case clusters, most intensely active at the time of investigation and due to the need for rapid response and rapid specimen transport to NPHL under cold chain conditions. Geographic restriction to Dar es Salaam limits our ability to comment on whether CVA24v was responsible for outbreaks in other regions of Tanzania, though phylogenetic relatedness to concurrent outbreaks in Kenya and Mayotte suggests regional circulation [31,32].

Beyond sampling representativeness, several additional limitations should be acknowledged:

i Incomplete genome recovery: We obtained partial CVA24v genomes (64–84% coverage) from two samples. This reflects challenges inherent in direct-from-specimen sequencing of RNA viruses with low viral loads.
ii Small number of sequenced genomes: Only two CVA24v genomes were successfully recovered. While this supports the detection of CVA24v during the outbreak, it is insufficient to establish definitive causation, precludes analysis of viral diversity or within-outbreak transmission dynamics, and prevents conclusions regarding the role of CVA24v in outbreaks in other regions or at earlier time points.
iii Lack of clinical data: Due to the urgent outbreak context, detailed clinical, demographic, and outcome data were not systematically collected.

Despite these limitations, this investigation achieved its primary objective: rapid genomic detection and phylogenetic characterization of CVA24v to support the public health response.

### Public health implications

The detection of CVA24v during this outbreak carries several public health implications. First, it supports evidence-based messaging around enterovirus conjunctivitis prevention (hand hygiene, avoiding shared towels and fomites) for future outbreaks. Second, it establishes a genomic baseline: NPHL now maintains CVA24v-specific PCR capacity and sequencing protocols for rapid deployment in future suspected AHC events. Third, this study contributes the first genomic data from Tanzania to global CVA24v surveillance; prior to this work, no CVA24v sequences from Tanzania were publicly available. Our data have been deposited in NCBI SRA (BioProject PRJNA1155894)

## Conclusion

This outbreak investigation reports the first genomic detection of CVA24v in Tanzania, identified during an acute hemorrhagic conjunctivitis outbreak in Dar es Salaam between January and February 2024. CVA24v was detected by genomic sequencing in two of four sequenced EV-positive samples from a convenience sample of 25 cases. While these findings are consistent with CVA24v as a likely contributor to the outbreak, the limited genomic coverage and small number of sequenced samples preclude definitive causal attribution. Phylogenetic analysis placed the Tanzanian sequences within a clade of contemporaneous CVA24v strains from East Africa and the Southwest Indian Ocean region, suggesting shared regional circulation.

This study provides the first genomic characterizations of an enterovirus detected during an AHC outbreak in Tanzania, contributing CVA24v sequence data from an under-represented region to global surveillance. The etiology of prior Tanzanian AHC outbreaks remains undetermined. Future investigations should aim to sequence a greater proportion of EV-positive samples and include broader geographic sampling to strengthen etiologic inferences.

Genomic epidemiology is a valuable tool for outbreak response, enabling rapid pathogen detection and phylogenetic contextualization even with limited samples and incomplete genome recovery. African public health laboratories should continue to strengthen capacity for enterovirus surveillance and genomic characterization of emerging disease threats, with investment in broader sampling frameworks to support stronger etiologic conclusions in future outbreak investigations.

## Supporting information

S1 Fig Comparison of phylogenetic trees: Full-length genome vs. VP1 sequences. Maximum likelihood phylogenetic trees inferred from full-length genome (left) and VP1 sequences (right) of [virus] isolates collected from [geographic region/outbreak]. Identical tree topology confirms phylogenetic signal consistency.
(TIF)
