# Supplementary figures and images for: Detection of Coxsackievirus A24v during an acute haemorrhagic conjunctivitis outbreak in Dar es Salaam, Tanzania, January-February 2024
Source: PLoS One. 2026 Jun 25;21(6):e0352698. doi: 10.1371/journal.pone.0352698 (PMC13298775; doi:10.1371/journal.pone.0352698)

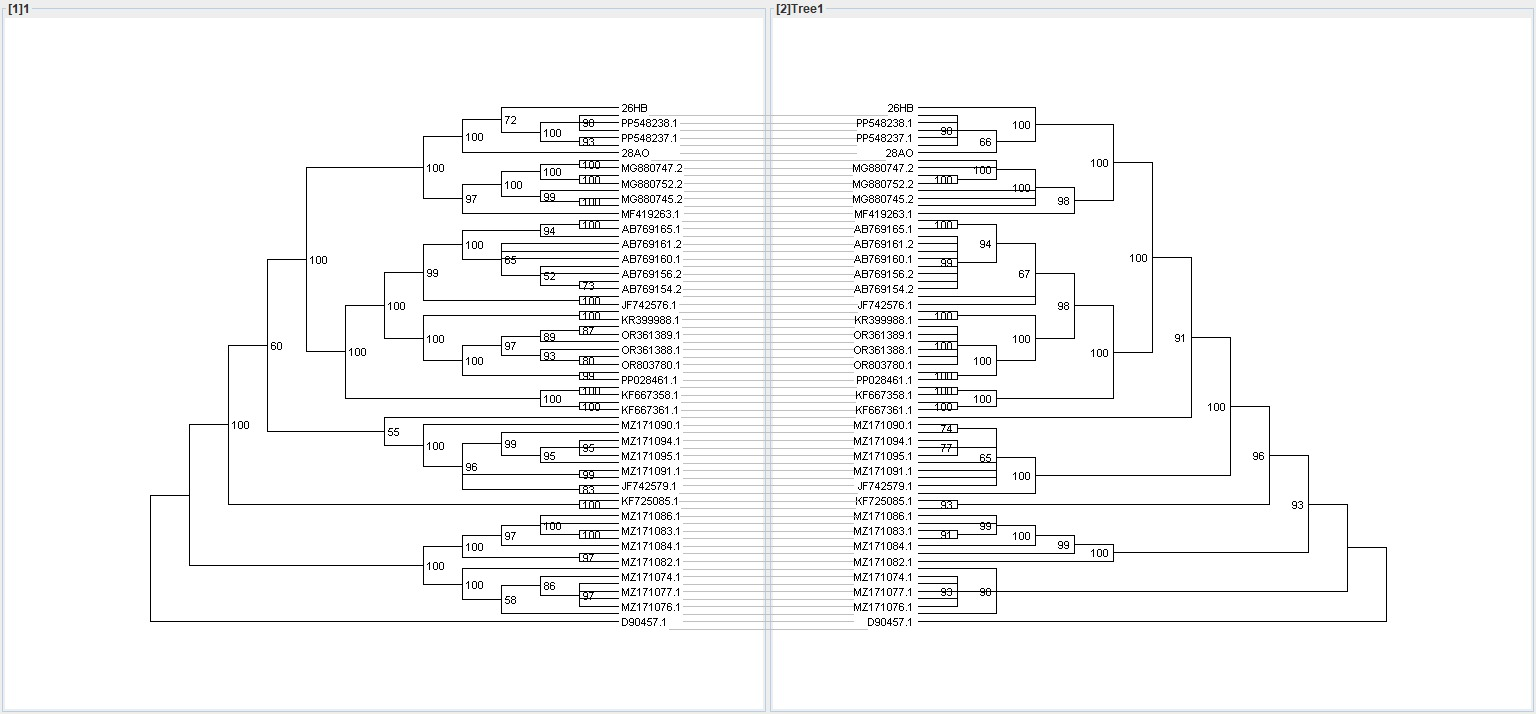

Supplement: S1 Fig — Maximum likelihood phylogenetic trees inferred from full-length genome (left) and VP1 sequences (right) of [virus] isolates collected from [geographic region/outbreak]. Identical tree topology confirms phylogenetic signal consistency. (TIF) [file pone.0352698.s001.tif]
